# 3D microscopic reconstruction of pearls using combined optical microscopy and photogrammetry

**DOI:** 10.1038/s41598-024-64680-7

**Published:** 2024-06-20

**Authors:** Paul-Emmanuel Edeline, Mickaël Leclercq, Jérémy Le Luyer, Arnaud Droit, Sébastien Chabrier

**Affiliations:** 1 Département de médecine moléculaire, Faculté de Médecine, Université Laval, Québec, G1V 0A6 Canada; 2 Géopole du Pacifique Sud, Université de Polynésie Française, 98704 Faa’a, Tahiti, Polynésie Française France; 3 Ifremer, IRD, Institut Louis-Malardé, Université de Polynésie Française, EIO, 98719 Taravao, Tahiti, Polynésie Française France; 4 Ifremer, CNRS, IRD, LEMAR, Plouzané, Université de Brest, 29280 Brest, France

**Keywords:** Tahitian pearls, Photogrammetry, Structure from motion, Optical microscopy, 3D, Biomineralization, Microscopy

## Abstract

In this study, we introduce an affordable and accessible method that combines optical microscopy and photogrammetry to reconstruct 3D models of Tahitian pearls. We present a novel device designed for acquiring microscopic images around a sphere using translational displacement stages and outline our method for reconstructing these images. We successfully created 3D models of two individual pearl rings, each representing 6.3% of the pearl’s surface. Additionally, we generated a combined model representing 10.3% of the pearl’s surface. This showcases the potential for reconstructing entire pearls with appropriate instrumentation. We emphasize that our approach extends beyond pearls and spherical objects and can be adapted for various object types using appropriate acquisition devices. We provide a proof of concept demonstrating the feasibility of 3D photogrammetry using optical microscopy. Consequently, our method offers a practical and cost-effective alternative for generating 3D models at a microscopic scale, particularly when detailed internal structure information is unnecessary.

## Introduction

Cultured pearls from *Pinctada margaritifera* (Linnaeus, 1758) are a key economic asset for French Polynesia. In 2021, production reached 10 million pearls, accounting for almost half of French Polynesia’s export revenue^1^. This industry employs over 3000 individuals, mainly in Tuamotu and Gambier islands. It generates an annual revenue of around 44 million $US. To maintain pearl market integrity, every pearl undergoes a rigorous quality assessment, and only those meeting specific standards are authorized for sale. To date, the majority of pearl assessments have relied on manual expertise, a process susceptible to external factors. Implementing new methods to automate pearl grading would be a valuable asset for the pearl industry, enhancing the accuracy of assessments and pearl unique identification. Furthermore, there has been intense research aimed at developing non-destructive methods to trace top-grade gems with the goal of improving production sustainability by achieving pearl certification. In this paper, we investigate the potential of utilizing optical microscopy to extract surface defects and aragonite overlying fronts visible on pearls at microscopic scale, which could ultimately enable pearl quality classification. To achieve this, we propose a 3D reconstruction method of the pearl surface at a microscopic scale, using photogrammetry, which could subsequently be employed for classification purposes.

The study of pearl formation and structure frequently involves imaging methods, with Scanning Electron Microscopy (SEM) and Transmission Electron Microscopy (TEM) being fairly common^2–5^. While these methods provide precise microstructural insights and aid in characterizing the formation and various deposits (calcite, aragonite) within pearls, they can be destructive to the pearl or financially prohibitive, limiting their widespread applicability. Therefore, this article proposes the use of an optical microscope to comprehensively characterize the surface of spherical or near-spherical pearls, offering an accessible solution that could be adopted on a larger scale. Despite the method’s inherent loss of precision compared to SEM/TEM, our findings illustrate that there is still a significant amount of information available for study at this scale and level of detail.

Optical microscopy on pearls has seen limited application in the literature^6,7^. Despite the challenges in acquiring and handling images in this context, various techniques are available for creating a 3D model from 2D microscopic images. These methods, including approaches such as image stitching^8–10^ and photogrammetry^11,12^, will be examined in this paper.

Photogrammetry is an optical technique used for reconstructing 3D models of objects from images taken at different viewpoints. While widely employed on a large scale, notably with drones, it is increasingly utilized for the reconstruction of small objects or at close-range^13,14^. It has also demonstrated its relevance at submillimetre-scale^15^ and finds primary application in archaeological studies^16–18^. However, photogrammetry is rarely applied to microscopic images due to the challenge of identifying matching points caused by the shallow depth of field inherent in such images. To address this, focus stacking can be used. It is an image processing technique that combines multiple images at different focal lengths to produce a single image with greater depth of field. Recent studies have confirmed its compatibility with photogrammetry^19,20^, thus paving the way for microscopic reconstruction through photogrammetry. Nonetheless, it is important to exercise caution with focus stacking, as the altered perspective it introduces may not always integrate smoothly with photogrammetry^21,22^.

In this paper, we investigate the potential of utilizing optical microscopy to ultimately classify pearl quality based on surface defects and aragonite overlying fronts visible on pearls at a microscopic scale. Our approach involves creating a 3D model of the pearl’s surface at this microscopic level using photogrammetry, a technique that could be further applied for classification purposes. For this study, we developed a specific device that automatically captures images around the pearl along a single axis with a fixed optical microscope, resulting in a comprehensive ring of images around the pearl. We detail the entire process, from initial image capture to the processing steps, leading to the successful 3D modeling of pearl rings using only optical microscope images. To validate our approach, we conducted tests on artificial 3D models, including a moon model. This simulation replicated a microscopic acquisition at the same scale and in the same manner as with our pearl, resulting in a successful 3D reconstruction of the entire moon. This serves as a proof of concept for the feasibility of this type of reconstruction, and our setup will be adjusted to enable broader acquisition for reconstructing the entire pearl.

The perspectives of obtaining such a model are multiple. Firstly, it will facilitate a more detailed analysis of the outer aragonite layers and defects surrounding the pearl, potentially leading to automated classification based on these features. Additionally, our process is not confined to pearls alone; it can be adapted for various other objects with suitably modified acquisition devices, enabling precise and affordable 3D reconstruction at a microscopic scale.

## Material and methods

In this section, we outline our comprehensive methodology for the 3D reconstruction of a ring around a pearl. The first part introduces our custom-designed acquisition system, specifically developed for this experiment. The second part describes an automated acquisition process, including all the necessary steps in image processing that result in the final images. In the third part, we focus on the reconstruction technique we employed to assemble acquired images into a 3D model. This part also includes the presentation of an artificially created 3D model of the moon, used as a simulated model to test the entire process and establish a baseline for accuracy. Overall, our approach provides a full pipeline, spanning from acquisition to processing, leading to a 3D model from acquired optical microscopy images.

### Microscopic acquisition system

**Figure 1:**
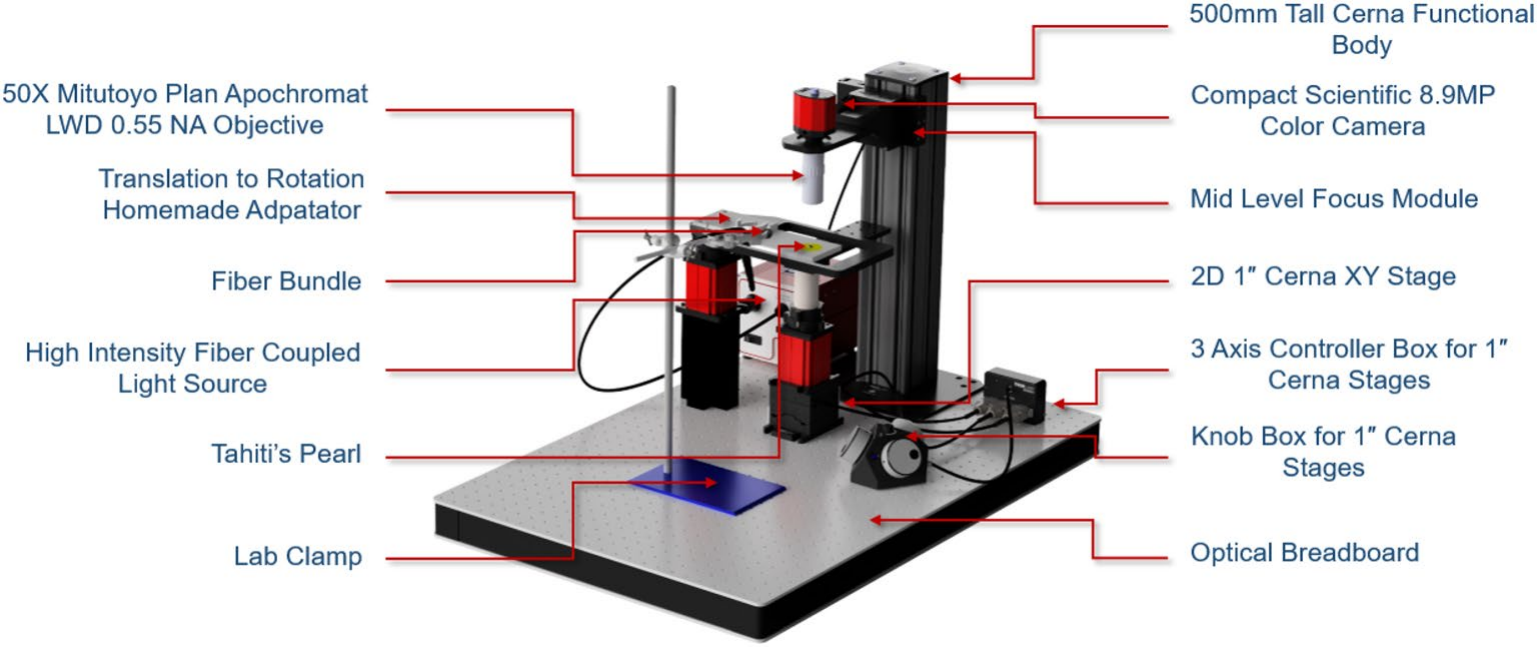
Full representation and description of the microscopic acquisition device.

Within the scope of this project, a specialized microscopic device was designed and implemented at the University of French Polynesia, using Thorlabs instruments. The entire setup is illustrated in Fig. 1. It features a Kiralux 8.9 MP CMOS Compact Scientific Camera (Sensor size: 14.131 mm  $\times$  7.452 mm), connected to interchangeable Mitutoyo Apochromatic objectives, which allow for 20$\times$ or 50$\times$ magnification. Both the camera and objectives are attached to a motorized focusing module with a 1^″^ (25.4 mm) travel range. Although supplementary objectives were at our disposal to achieve magnifications ranging from 100$\times$ to 200$\times$, we ultimately opted for a 50$\times$ magnification. This decision was made because a magnification of 20$\times$ was not precise enough, while magnifications of 100$\times$ and higher resulted in excessive illumination and vibration issues.

Additionally, the device is equipped with a 2D 1^″^ Cerna XY Stage, a two-axis translation stage (velocity : 7 mm s^-1^) that allows the movement of the pearls under examination. However, the limited range of motion in each axis of the translation stage prevented a full rotation around the pearl in both directions. Indeed, the maximum effective displacement range accessible through the native API for one plate was 17 mm, whereas the circumference of an 8 mm pearl is 25 mm. Consequently, we decided to align both translation stages along the same axis. This configuration limited movement to a single direction, yet it allowed for the successful capture of full rings around the pearl in that direction.

The translation stage and motorized focusing module were connected to a Knob Box for 1^″^ Cerna Stages, which provides manual control over focus and movement. Additionally, they were linked to the MCM3000 Thorlabs 3-Axis controller, allowing for automated control of the various movements via a computer interface.

Light management was handled by the Thorlabs OSL2 High-Intensity Fiber-Coupled Illuminator and its associated fiber bundle, along with the Thorlabs OSL2COL Collimation package. These components were manually adjusted using a lab clamp to ensure that the light falls as evenly as possible on our pearls, minimizing the reflective impact of the pearl’s surface. To have an optimal illumination, it was essentiel to manage four interrelated parameters: the intensity of the light source, the distance and angle from the light source to the pearl, and the exposure settings of the CMOS camera. The challenges arising from illumination are illustrated in Supplementary Fig. 1. This figure compares full images taken under different lighting conditions, before images were cropped to retain only the areas with sufficient illumination. The entire device were mounted on a robust optical breadboard to minimize vibrations.

**Figure 2:**
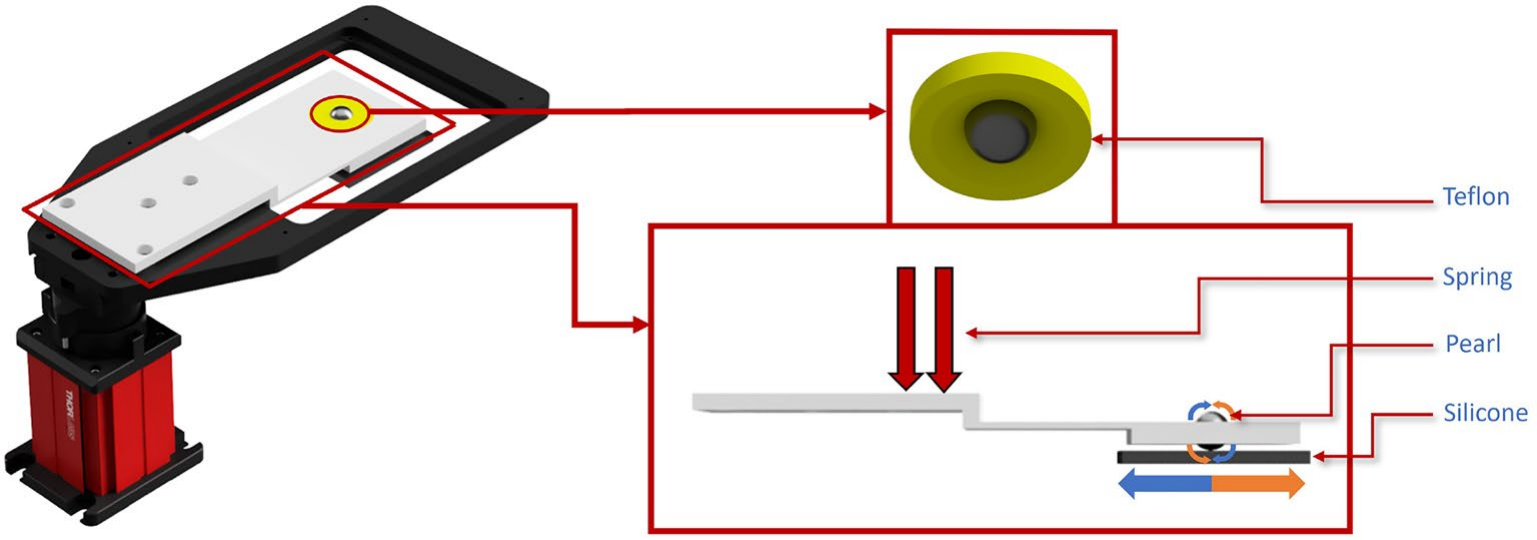
Rotation adaptor for translation. Pearl was positioned on a silicone plate and fixed with Teflon under the microscope, using an adjustable spring for optimal support force.

To enable the servo acquisition of the entire surface of the pearl using only a fixed camera and translation stage, we developed a device that converts translational motion into rotational motion. This translation-to-rotation adapter, depicted in Fig. 2, has a beveled shape that offers upper support for the pearl while leaving sufficient space for image acquisition. It was made of Teflon, selected for its exceptionally low friction to avoid hindering rotation. Below the pearl, a silicone plate, chosen for its adhesive properties, were attached and connected to the translation stage.

This entire device, by utilizing the downward pressure exerted by the pearl on the adhesive silicone, facilitated the conversion of the translational movement of the silicone plate into rotational motion for the pearl along one axis. Consequently, this allowed for the acquisition of complete rings around the pearl. However, due to friction, there was a movement loss of approximately 25%. Indeed, to complete a full ring, a pearl with an 8 mm diameter required roughly 32 mm on the translation stages, compared to the theoretically expected 25 mm.

### Acquisition process

After precisely positioning the study pearl and ensuring optimal illumination within the device, the image acquisition could begin. To automate the acquisition of a ring of images around the pearl, we developed an interface using Matlab. The complete interface is shown in Supplementary Fig. 2. The various steps of this program can be summarized as follows:

1. Establishing the connection between the computer, camera, and motion controller.
2. Configuring the camera settings, which include exposure, color balance, gain, and black level adjustments.
3. Setting displacement parameters, such as travel limits, and automating the movement of the translation stages between each acquired frame.
4. Specifying image processing settings, which involve defining a region of interest, setting up autofocus, implementing focus stacking, and converting input data from the CMOS camera into an RGB image, through the native Thorlabs API.

Once all these parameters, that are available in Supplementary Fig. 2, have been selected, the initial focus and position of the pearl were manually set. The acquisition process then automatically captured all the images forming a ring around the pearl and progressively saved the processed images until the acquisition is complete. The dimensions of the saved images, relative to the original dimensions of the camera acquisition, are displayed in Supplementary Fig. 3. Taking into account the travel times imposed by our equipment, the communication between the computer and the equipment, as well as the autofocus and focus stacking required at each travel step, a full acquisition for a ring typically takes an average of 120 min.

After evaluation of various methods and metrics, we chose to use numerical autofocus with the *fmeasure* function in Matlab^23^, employing squared gradient metrics (GRAS), which proved to be one of the most suited for our images, as depicted in Supplementary Fig. 4. For focus stacking, we used Helicon Focus^24^, which was the most efficient for our images.

### Image reconstruction

After successfully acquiring images of our pearl, our next goal was to reconstruct these images into a 3D structure. We first assessed the feasibility of this reconstruction using a simulated model. This approach allowed for a direct comparison between our reconstructed and simulated models. To this end, we created a 3D model of the moon using Blender^25^ software, simulating image acquisition at a scale identical to that of our pearl images. The moon was chosen for its spherical shape and distinctive features visible at this scale, unlike other models such as Earth. The spherical model of the moon exhibited the most analogous features observable on pearls, at a comparable scale, taking into account the ratio between feature scale and sphere diameter. The photogrammetric reconstruction, carried out with Agisoft Metashape^11^, is shown in Supplementary Fig. 5. As the reconstructed 3D model closely resembled our simulated model, we proceeded with the reconstruction of the pearl images.

To successfully reconstruct a pearl ring, our process involved several critical steps. We had to adjust the camera calibration parameters such as focal length from their default settings, a necessity brought on by the focus stacking in our images. Additionally, we refined the photogrammetric process and the calculation of camera positions by employing a specific routine on an image dataset. This routine proved essential because, although we managed to reconstruct our moon dataset without it, the same method was ineffective for the pearl dataset, where the images were even more challenging to differentiate. The routine is as follows:

1. Calculate the actual displacement between each image pair using SIFT (Scale-Invariant Feature Transform) analysis to each set of matched points.
2. Convert the linear shift into a rotational shift, based on the assumption of the pearl’s spherical shape.
3. Create a specialized moon dataset from our simulated model, including images that align with the rotation positions determined from the shift.
4. Align the images from this specific moon dataset using Agisoft Metashape.
5. Determine and extract the camera positions from this alignment.
6. Import and apply these camera positions to the pearl reconstruction, using the original dataset.

This approach allowed us to take advantage of the relative simplicity of reconstructing our simulated model, which subsequently facilitated the reconstruction of the pearl using our collection of real images. At this point, the moon could be interchanged with any object, and we only take benefit from the reconstructed spherical model at the correct scale and focal length. The Metashape Python API was used to automate the optimization of camera calibration parameters, with a specific focus on the focal length. To determine the optimal focal length, we conducted several alignment trials. Screenshots were taken from various angles of each alignment. Metrics were then automatically extracted from these screenshots, leading to the empirical definition of the error measure:

1 $$\begin{aligned} E_{tot} = \frac{\epsilon }{\lambda _1} + \frac{E_{vertical}}{\lambda _2} + \frac{E_{horizontal}}{\lambda _3} \end{aligned}$$

In this equation, $\epsilon$ represents the ellipticity of the ring formed, determined by fitting an ellipse to the vertical view of the model. $E_{vertical}$ measures the distance from the start to the end of the ring in the vertical view, while $E_{horizontal}$ measures this distance in the horizontal view. Both distances are calculated using edge detection and contour analysis. The terms $\lambda _1$, $\lambda _2$ and $\lambda _3$ are normalization factors designed to ensure that each criterion is equally weighted. $\epsilon$ eliminates alignment that deviate from a ring shape, while $E_{vertical}$ and $E_{horizontal}$ more precisely discriminate a perfect ring from an incomplete or distorted one. The optimal parameters are those that correspond to the alignment and reconstruction process which results in the minimization of $E_{tot}$, the total error metric.

Furthermore, when the reconstruction process encountered challenges with image pairs not aligning automatically, despite apparent connections, manually adding markers was employed as a solution. These issues were specifically observed when the overlap was less than 65% between our images, due to a sliding phenomenon. Typically, re-running the alignment phase after these manual adjustments resolved the issue. The process of manually adding markers is illustrated in Supplementary Fig. 6.

**Figure 3:**
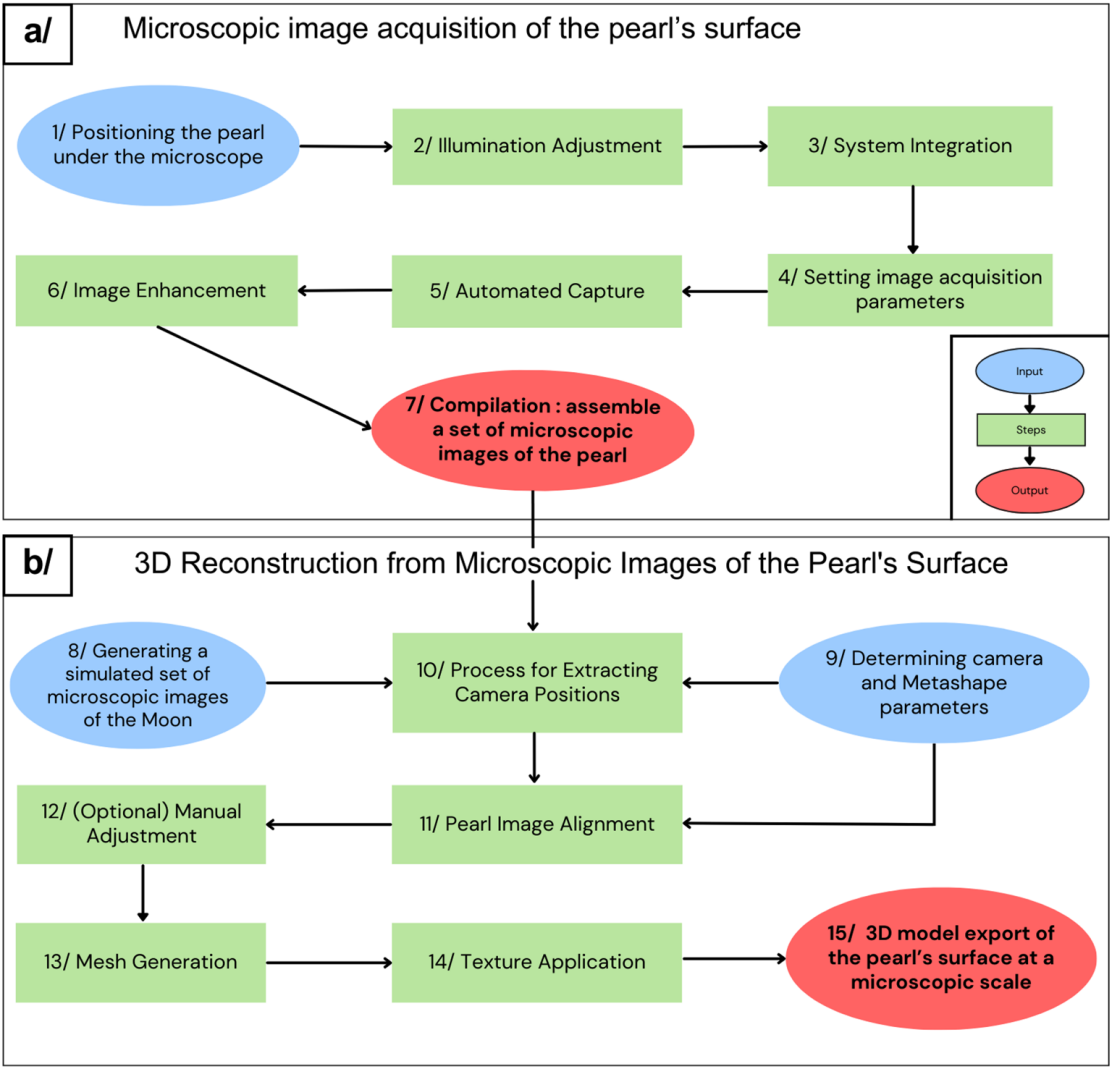
Complete overview of the data process, from pearl image acquisition to 3D reconstruction. (**a**) Microscopic Image Acquisition of the Pearl’s Surface. (**b**) Three-Dimensional Reconstruction from Microscopic Images of the Pearl’s Surface. The entire process, encompassing these various steps, is automated within a singular workflow using Matlab, Python, and Agisoft Metashape.

A comprehensive diagram is provided in Fig. 3, outlining all the steps from acquisition to reconstruction in our study. For an in-depth explanation of each steps, refer to Supplementary Note 1. The results from our 3D pearl reconstruction will be summarized and discussed in the "Results" section.

## Results and discussion

Our objective is to evaluate the precision of our reconstructed 3D model of the microscopic surface of the pearl. Additionally, we aim to determine the feasibility of applying the process of photogrammetry to optical microscopic images.

### Optical microscopy of a pearl’s surface

**Figure 4:**
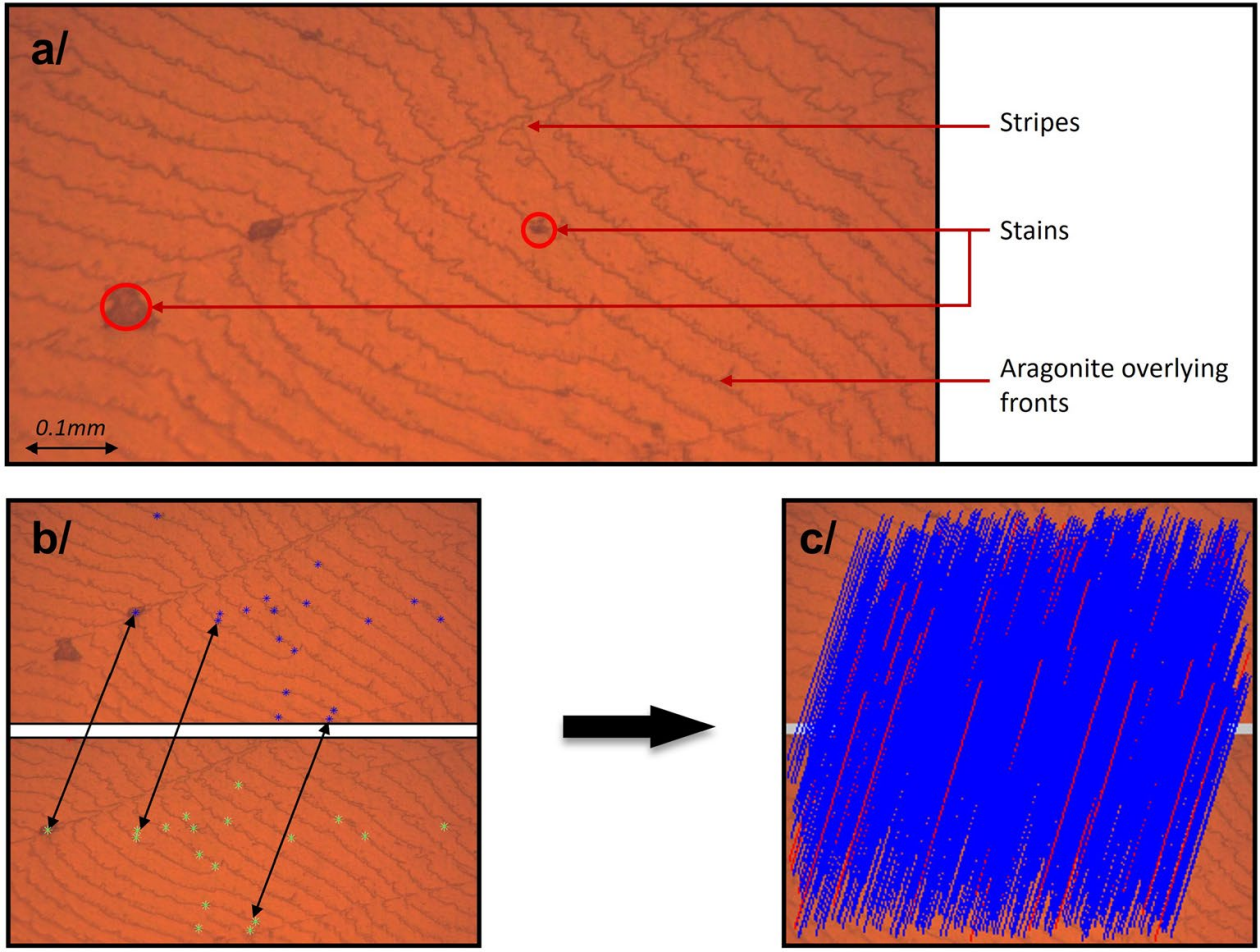
Images of a pearl’s surface under optical microscopy. (**a**) Annotated microscope image at 50$\times$ magnification, showing visible stripes, stains, and overlying aragonite fronts. (**b**) A depiction of selected SIFT descriptors, marked in green and blue on two distinct images shifted by 4^∘^. (**c**) The complete set of SIFT descriptors computed during the reconstruction process on the same two images, including their matches. Blue lines indicated matches used in the reconstruction, whereas red lines represent matches that were not used.

The microscopic images obtained in this study are displayed in Fig. 4. At 50$\times$ magnification, the primary features observed on the pearl’s surface include stripes, stains, and overlying aragonite fronts. Based on observation, the sizes of these features are as follows: 0.02 to 0.2 mm for strains, 0.005 to 0.01 mm for aragonite overlying fronts, and stripes ranging from 0.05 mm to encompassing the entire circumference of the pearl. Figure 4b highlights some SIFT descriptors calculated on two images of our dataset, separated by a 4^∘^   angle. This emphasizes the challenge of identifying corresponding points in our images, due to the limited number of distinctive features. Figure 4c displays the final SIFT descriptors and their matches, as identified during the reconstruction process using Agisoft Metashape. This figure demonstrates that the reconstruction process utilizes not only stripes and stains but every element in the images. In this specific example, blue lines represent matches between two descriptors that contribute to the reconstruction, while red lines indicate matches that are not used. Here, 1859 valid matches and 64 invalid matches are depicted.

All microscopic images were captured of a round Tahitian pearl, with its specific characteristics detailed in Supplementary Table 1. We reconstructed two different rings; our completed image dataset corresponding to approximately 10 and 10.5 megabytes, respectively. To acquire each ring, an acquisition time of 2 h was necessary. These feature an average overlap of 68% with a standard deviation of ± 5.8% for Ring 1, and an average overlap of 69.6% with a standard deviation of ± 6.9% for Ring 2. As a result, the reconstructed rings represent 6.3% of the pearl’s total surface area each. The notable standard deviation in overlap is due to a sliding phenomenon, caused by imperfections in either the pearls or the silicone plate. Since pearls are not perfectly spherical, this results in inconsistent movement around the entire circumference of the rings. These variations in image overlap present significant challenges during the reconstruction process, occasionally necessitating manual marker corrections on problematic images.

### 3D reconstruction of a pearl’s surface

Before adopting a photogrammetric approach for our 3D reconstruction, we initially implemented and evaluated image stitching algorithms^8–10^ on our images. However, these did not yield significant results, leading us to quickly transition to photogrammetry.

**Figure 5:**
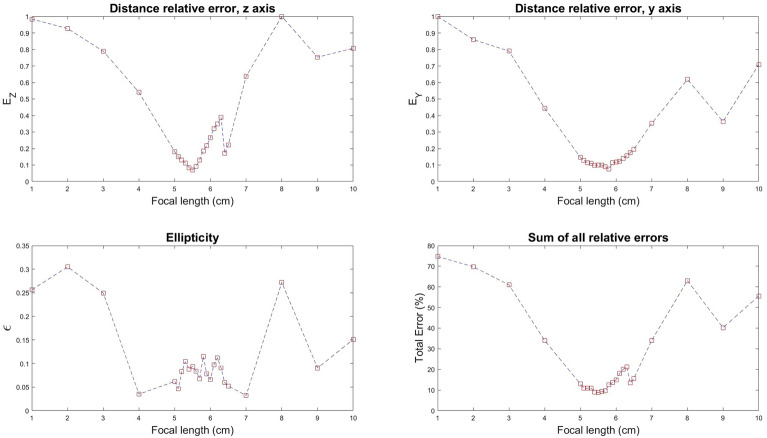
Measurement of alignment errors in relation to varying focal lengths. The calculated errors are detailed in the “Material and methods” section.

As detailed in the “Material and methods” section, we implemented several steps to enhance our reconstruction process. A crucial step was calibrating the focal length parameters to account for the focus stacking effect in our images. The outcomes, based on our empirical metrics, are shown in Fig. 5. We achieved optimal alignment with a focal length of 5.75 mm, an adjustment from the initial effective focal length of 4 mm of our optical system. Consequently, the final camera calibration parameters used in our reconstruction were a focal length of 5.75 mm and a pixel size of 3.45  $\upmu$m $\times$ 3.45  $\upmu$m. Another essential step for successful reconstruction involved importing camera positions before initiating the alignment phase, as previously described. Without this step of reconstructing camera positions, the alignment phase failed to align all images effectively, and the results of such misalignment are shown in Supplementary Fig. 7. Therefore, this step was crucial for the success of our reconstruction.

**Figure 6:**
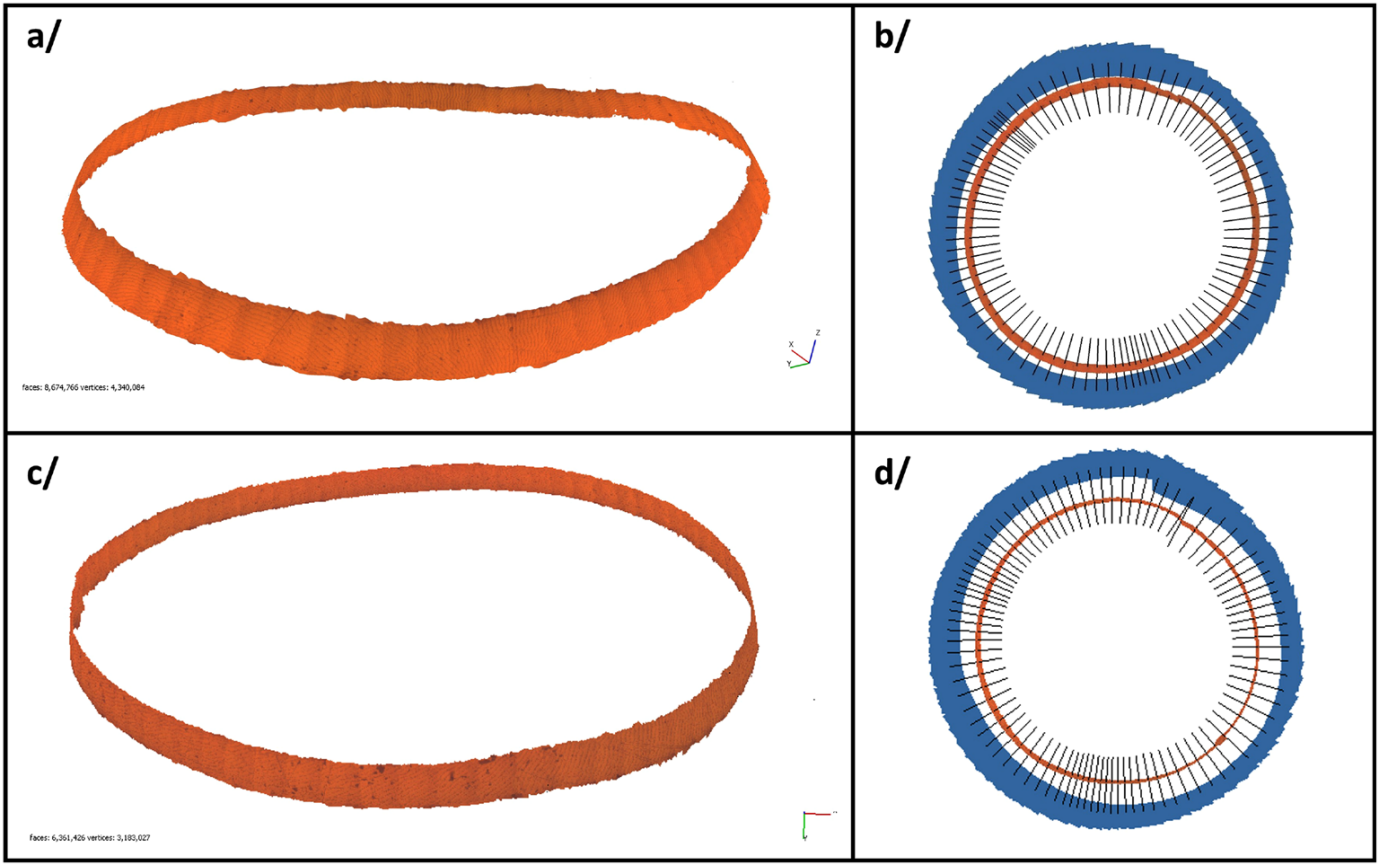
Reconstruction of the entire rings: (**a**) Ring 1: Textured model generated from images of our pearl’s surface. (**b**) Ring 1: Corresponding camera positions associated with the model. (**c**) Ring 2: Textured model generated from images of our pearl’s surface. (**d**) Ring 2: Corresponding camera positions associated with the model.

Through these steps, we successfully completed a 3D reconstruction of a microscopic surface section of two pearl’s rings, as shown in Fig. 6. In addition to the steps mentioned above, every parameter used in the reconstruction process, from the alignment phase to the application of texture, is detailed in Supplementary Fig. 8. To address the issue of insufficient overlap in two specific images for Ring 1 and Ring 2, manual marker corrections were applied during the alignment phases. The remainder of the process was automated using the Metashape Python API. The entire reconstruction procedure required approximately 1 h of computation time on an NVIDIA Quadro P400 GPU for each ring.

Following the successful reconstruction of our pearl images, it was essential to evaluate the accuracy of our models. Since no pre-existing model of a pearl surface was available for comparison, we developed our own metrics. We chose to compare the minimum and maximum diameters of both the pearl and our models. By knowing the width of the ring, which corresponds to the width of an image, we were able to set a scale for our models in millimeters and measure these diameters. The related calculations are detailed in Supplementary Note 2. We also calculated the ellipticity from these diameters to analyze the ring’s curvature. The results are presented in Tables 1, 2 and 3. The results were highly satisfactory, showing accuracies of 99.6% and 99.5% for the minimum and maximum radius comparisons, respectively, for Ring 1, and 99.0% and 99.7% for the minimum and maximum radius comparisons, respectively, for Ring 2. Additionally, the diameter measurements of the pearls were conducted on the entire pearl, not just on the specific ring segment we focused on. Consequently, it was expected that the comparison would not be exactly the same, which explains the significant difference in ellipticity: as anticipated, our rings are more circular compared to the theoretical ring formed by the maximum and minimum diameters of the full pearl, which has a higher ellipticity value.

**Table 1:** Calculated metrics to evaluate the accuracy of the reconstructed 3D model: Ring 1.

Metric	Measured value	Reconstructed value	Accuracy (% )
Min. Diameter (mm)	8.24 ± 0.02	8.27 ± 0.02	99.6
Max. Diameter (mm)	8.45 ± 0.02	8.41 ± 0.02	99.5

**Table 2:** Calculated metrics to evaluate the accuracy of the reconstructed 3D model: Ring 2.

Metric	Measured value	Reconstructed value	Accuracy (%)
Min. Diameter (mm)	8.24 ± 0.02	8.32 ± 0.02	99.0
Max. Diameter (mm)	8.45 ± 0.02	8.43 ± 0.02	99.7

**Table 3:** Calculated ellipticity to evaluate the accuracy of the reconstructed 3D models.

Metric	Measured value	Reconstructed value
Ring 1 Ellipticity	0.025 ± 0.003	0.013 ± 0.003
Ring 2 Ellipticity	0.025 ± 0.003	0.013 ± 0.003

**Figure 7:**
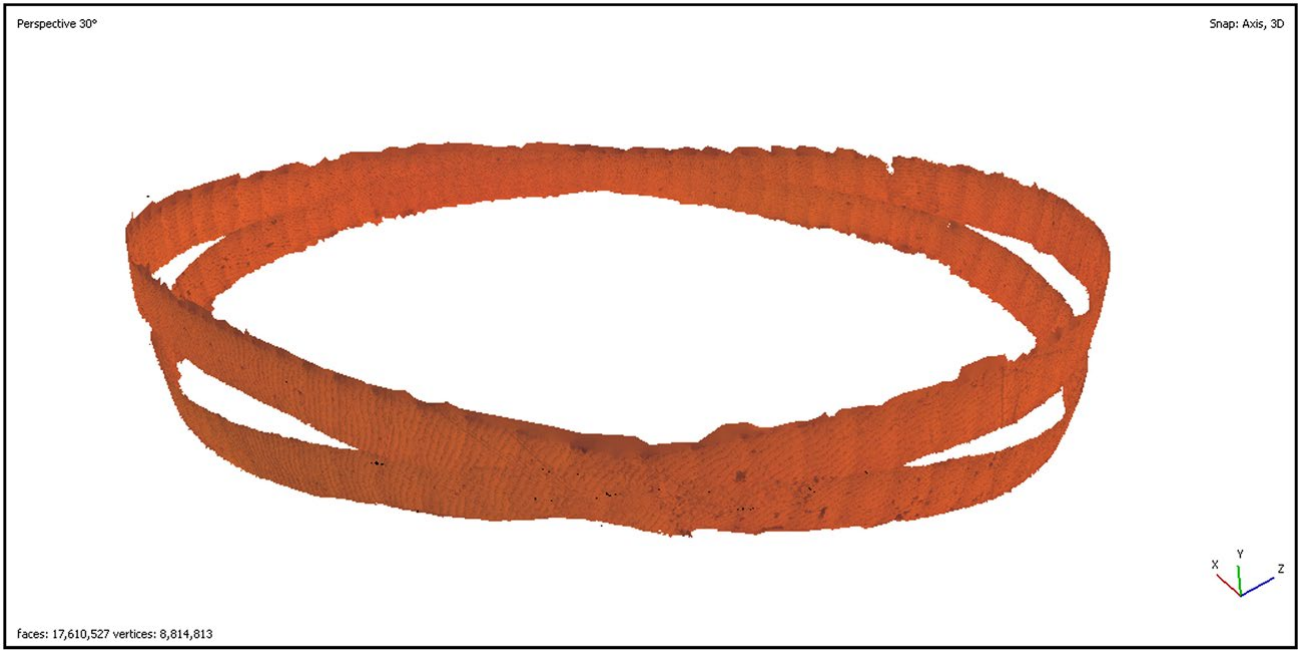
Combined reconstruction of both rings.

After reconstructing the two distinct rings separately, we attempted to reconstruct them together, despite the suboptimal overlap between the rings. Although challenging, the reconstruction was successful using the same method as before, across the 182 images. This process took up to 3 h, and the final model’s surface was estimated to represent 10.3% of the pearl’s surface, as calculated using MeshLab^26^. The full model is depicted in Fig. 7, and an example of an area where the rings intersect is illustrated in Supplementary Fig. 10. Despite some small defects in the reconstruction, this demonstrates the feasibility of reconstructing the entire surface of the pearl using multiple successive rings.

## Limitations

This study faced several challenges, with one of the most significant being the sliding phenomenon between the pearl and the silicone, which limited our ability to reconstruct many rings. Specifically, sliding in the direction perpendicular to our travel path resulted in a loss of overlap between two images, making our dataset unusable. Even sliding parallel to our direction could quickly lead to insufficient overlap. This issue primarily arose from the limitations of our displacement stages’ travel range, necessitating their use to the maximum extent. This, coupled with friction issues, hindered our ability to adjust parameters to achieve a full ring with a larger and more efficient overlap. Software constraints limited our maximum displacement range to approximately 34 mm, although our calculations indicated that at least 32 mm was necessary to successfully reconstruct an entire ring. Consequently, we had to find a balance between achieving sufficient overlap and managing friction. Smaller displacements improved overlap but also increased friction, necessitating a larger displacement range.

Another significant challenge we faced was the absence of a reference pearl for comparison. In addition to pearls, we were unable to identify any other suitable object that could be integrated into our experimental setup for validation purposes. Consequently, we had to rely on the measurements available to us for validating our model. While we endeavored to make the best use of the available resources, we acknowledge the limitations of this approach. The lack of a standard model for comparison meant that our validation process was restricted, depending on the specific characteristics and measurements of the pearls we used, rather than on a more broadly applicable standard.

Finally, our approach is optimized for spherical or quasi-spherical pearls. To study pearls of more varied shapes, both our device and processing methods would need to be adapted. This limitation underscores the need for caution in generalizing our findings and highlights the specificity of our experimental conditions.

## Future perspectives

Despite these limitations, we remain confident in the validity of our 3D model and see great potential in achieving a full reconstruction of a pearl, as evidenced by our reconstruction of two rings in Fig. 7. We strongly believe that this can be accomplished by successfully acquiring multiple rings. By making necessary adjustments to our displacement stages and following the same process, we aim to acquire about 30 successive rings. This should provide enough overlap on both the x and y axes, theoretically enabling us to reconstruct the entire pearl using an estimated 3000 images. Achieving a complete 3D model of the pearl’s surface would open up new paths for studying its various features. This includes exploring the relationship between a pearl’s attributes (such as quality, luster, and shape) and its surface elements (like stains, stripes, and aragonite overlying fronts). Such studies could potentially lead to automated classification of certain attributes, and offer a novel tool for examining pearls and their mineral structures. This approach would be significantly more cost-effective and non-invasive compared to Scanning Electron Microscope (SEM) or Transmission Electron Microscopy (TEM) studies.

Furthermore, we believe that this methodology could be applied to a wide range of objects in various research contexts, particularly due to its cost-effectiveness. Although our experimental setup is currently specialized for pearl analysis and would require modifications for other applications, the fundamental principles of the reconstruction process should be broadly applicable. With minimal adjustments, this technique could be effectively adapted for reconstructing other objects using optical microscopy. This versatility not only makes it a valuable tool in various scientific fields but also enhances its potential for widespread use, especially in studies where budget constraints are a significant consideration. The ability to apply this method to diverse objects could lead to new discoveries and advancements in fields such as materials science, biology, and archaeology, where detailed surface analysis is crucial.

## Conclusion

In this paper, we established that optical microscopy is proficient at generating high-quality images of the pearl surface, revealing valuable details such as nacre surface deposits and defects, invisible to the naked eye. We then broadened its application, moving beyond single images to capture multiple views all around the pearl, in order to obtain a 3D reconstruction of its surface. We introduced a specialized system designed to convert translational motion into rotational motion, enabling effective acquisition of microscopic images of a pearl’s surface. Subsequently, our results have successfully demonstrated the reconstruction of a 3D model representing 10.3% of the pearl’s surface. This achievement underscores the potential for photogrammetric reconstruction using optical microscopy images. A crucial aspect of our methodology involved making precise adjustments to address the inherent limitations of depth of field in these images. This included the use of techniques such as focus stacking and its implications on the reconstruction phase.

Looking ahead, this research shows promises for advancing the field of pearl studies. It establishes the groundwork for more comprehensive examinations of pearls, potentially linking surface characteristics and broader attributes such as quality and luster. Pending more detailed and affirmative studies concerning the correlations between surface characteristics and the attributes of pearls, these findings could potentially lead to the development of automated classification systems for pearl attributes based on microscopic images.

Furthermore, the implications of this work extend beyond pearls, suggesting a wider applicability for conducting detailed, cost-effective studies across various fields. The flexibility of our approach allows for its potential application to a broad range of objects, offering an economical solution for in-depth surface analysis. This paper presents a novel method for exploring small-scale structures, providing a more accessible alternative to more expensive techniques.

In conclusion, our study contributes to the field of pearl research by introducing an affordable device for accessing microscopic-scale information on the surface. Additionally, we pave the way for innovative and cost-effective methods in microscopic imaging and 3D reconstruction for surface studies. This has the potential to significantly benefit researchers operating with limited resources, opening up new avenues for exploration and discovery across various scientific disciplines.

### Supplementary Information

Supplementary Information.

## Data Availability

The data supporting the findings of this study, along with the images used and the 3D models computed, are available online at https://doi.org/10.5281/zenodo.10472502. Without an Agisoft Metashape license, models are still viewable using the free Agisoft Viewer software.

## Code availability

The code for acquiring and processing images, as well as for creating the 3D models, is available online at https://doi.org/10.5281/zenodo.10472502. Any additional minor codes used in our study can be obtained from the corresponding author upon reasonable request.
