# Supplementary material for: 3D microscopic reconstruction of pearls using combined optical microscopy and photogrammetry
Source: Sci Rep. 2024 Jun 20;14:14180. doi: 10.1038/s41598-024-64680-7 (PMC11186833; doi:10.1038/s41598-024-64680-7)
Supplement: Supplementary file 1 — Supplementary Information. [file 41598_2024_64680_MOESM1_ESM.pdf]

Supplementary Materials

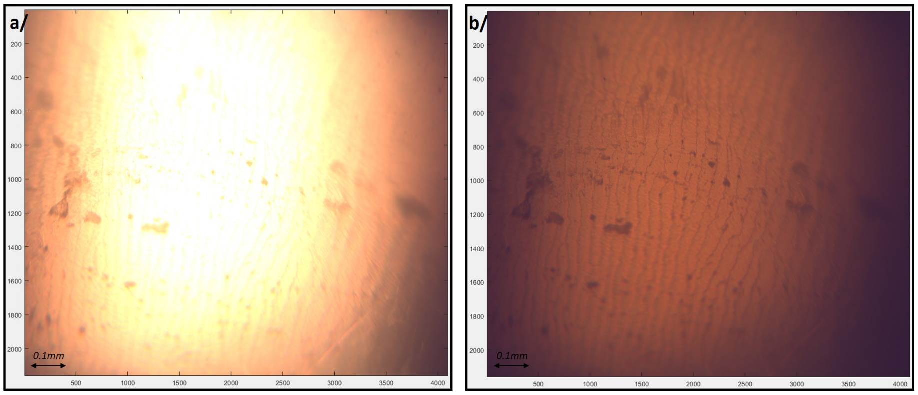

Figure 1: Pearl image acquired using an optical microscope: **a/** With suboptimal illumination parameters, featuring light directed perpendicularly to the pearl. **b/** With optimal illumination, where light is directed parallel to the pearl. This highlights the importance of careful light management in our context.

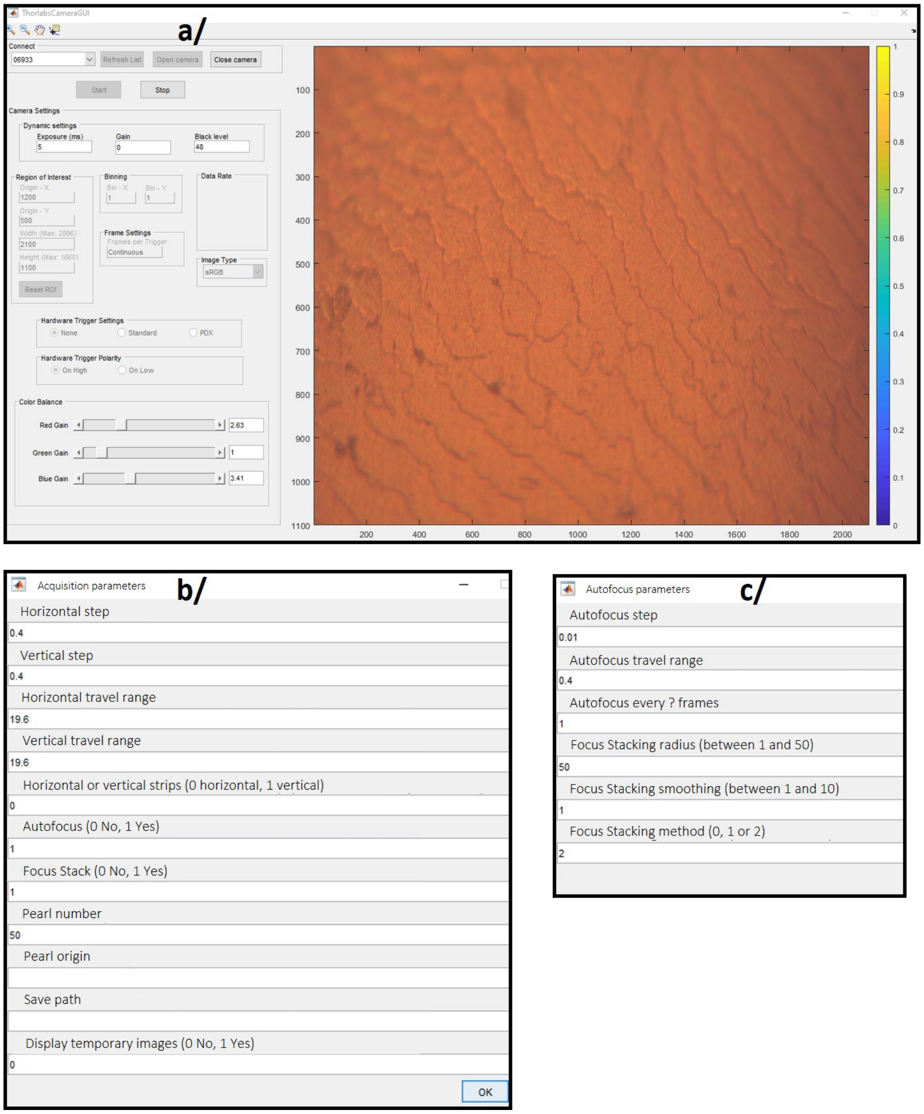

Figure 2: Full Interface for Automated Image Capture.
a/ Camera Settings (including exposure, cropping, black level, etc.). **b/** Displacement Parameters (such as step size and range). **c/** Autofocus and Focus Stacking Settings (covering focus range and steps). All these parameters were chosen empirically.

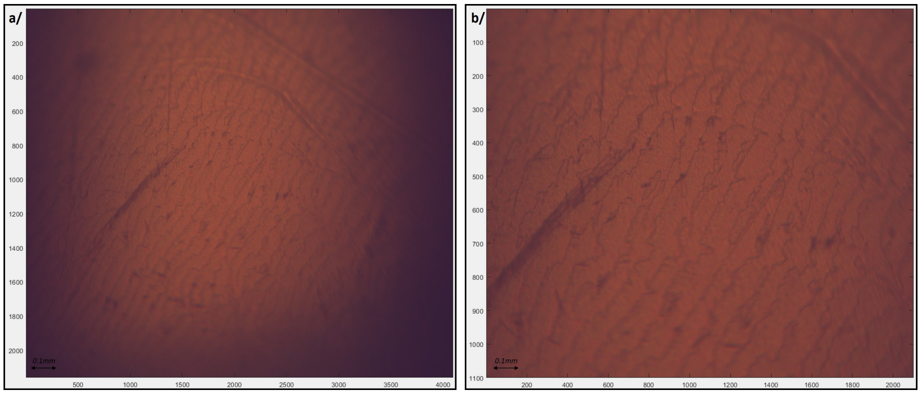

Figure 3: Pearl image acquired using an optical microscope. **a/** Before cropping to an optimal illumination area, 4096*2160 pixels. **b/** After cropping to an optimal illumination area, 2100*1100 pixels. This underscores the fact that, due to the pearl's curvature, the area that can be optimally illuminated is quite limited.

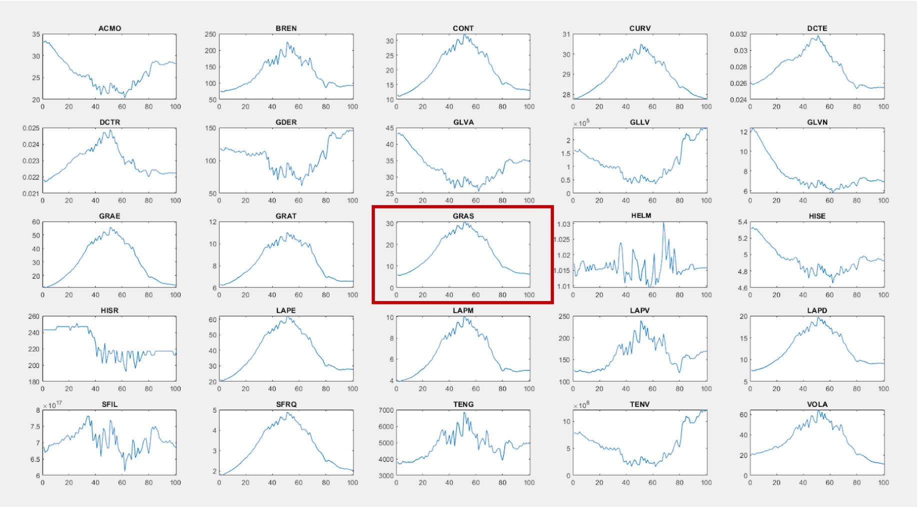

Figure 4: Various metrics were evaluated using the fmeasure function in Matlab. The abscissa represents different images captured at the same point but with varying focus levels. GRAS was chosen for autofocus implementation, as it effectively identified the sharpest images in our test dataset.

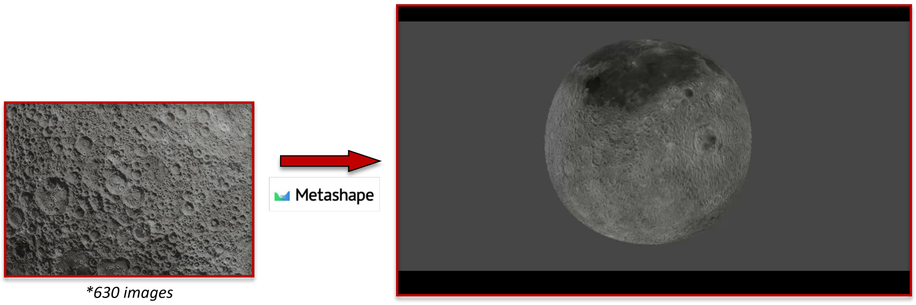

Figure 5: Photogrammetric reconstruction of blender-made moon through Metashape. The full moon was reconstructed, through multiple successive rings reconstruction.

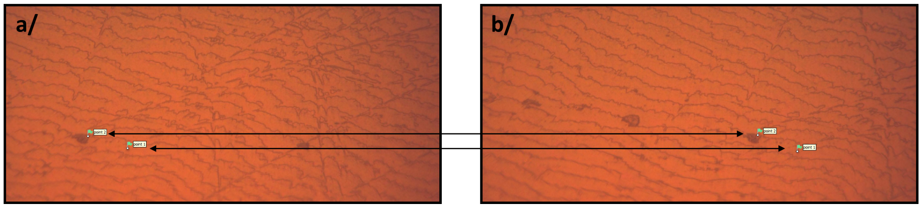

Figure 6: Example of manually adding markers: **a)** Image 1, with two markers added manually. **b)** Image 2, showing the placement of the corresponding two markers from Image 1. This method is employed when successive images fail to reconstruct, often due to insufficient overlap between them. It is done through the 'Add Markers' feature in Agisoft Metashape Professional Edition.

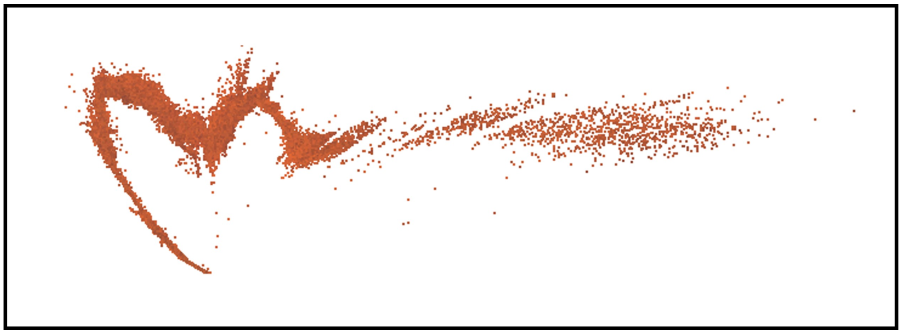

Figure 7: Example of alignment without importing camera positions. This underscores the fact that, without this step in our process, we encountered difficulties in achieving correct alignment between our images, which is crucial for successfully creating a 3D model of our pearl.

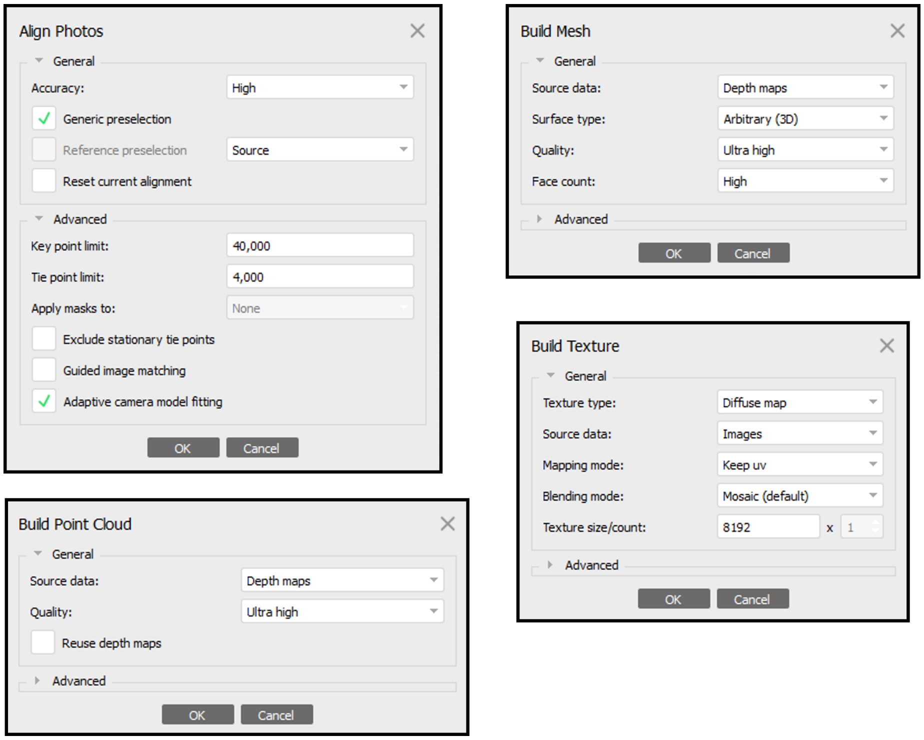

Figure 8: Final alignment parameters. Alignment was made using generic preselection, high quality, and aligning images one-by-one, using metashape python API. Next steps were done using Agisoft Metashape Professional interface. **a/** Dense Cloud generation parameters. **b/** Mesh generation paremeters. **c/** Texture application parameters.

**Class**	B
**Shape**	Round
**Luster**	Good
**Color Intensity**	Medium
**Weight** (g)	1.00681
**Min. Nacre Thickness** (mm)	1.18
**Max. Nacre Thickness** (mm)	1.31
**Min . Diameter** (mm)	8.452
**Max. Diameter** (mm)	8.242

Table 1: Pearl attributes. Classification made by *Direction des Ressources Marines, Tahiti.*

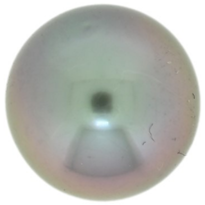

Figure 9: Photography of the pearl used for acquisition, taken with a Nikon D7100.

Supplementary Note 1 : Diagram Description

From Fig. 3 : Complete overview of the data process, from pearl image acquisition to 3D reconstruction. Details of each steps :

- 1/ Preparation: Positioning the pearl under the microscope.
- 2/ Illumination Adjustment: Modifying the light intensity and brightness for optimal visibility.
- 3/ System Integration: Connecting the microscope to the computer, including the camera and motorized stage.
- 4/ Configure the image acquisition parameters.
- 5/ Automated Capture: Initiating automatic image capture process.
- 6/ Image Enhancement: Applying digital filtering to the captured images.
- 7/ Compilation: Assembling our dataset of microscopic images of the pearl.
- 8/ Simulation Creation: Generating a simulated dataset of microscopic Moon images, through Blender.
- 9/ Parameter Configuration: Setting the camera and Metashape software parameters.
- 10/ Process for Extracting Camera Positions : a) Determining the displacement between each image and converting these into angular coordinates θ and ϕ. b) Image Subset Creation: Generating a modified set of Moon images representing only the calculated pearl positions. c) Image Alignment in Agisoft Metashape: aligning the selected Moon images. d) Camera Position Extraction : Retrieving camera positions from the alignment in Metashape format.
- 11/ Pearl Image Alignment: Aligning the pearl images using the imported camera positions.
- 12/ Manual Adjustment (Optional): Adding markers manually to refine alignment if necessary.
- 13/ Mesh Generation: Creating a digital mesh of the pearl's surface, from the alignment.
- 14/ Texture Application: Adding surface texture to the generated mesh.
- 15/ 3D Model Export: Producing and exporting the final 3D model of the pearl's surface at a microscopic scale.

Supplementary Note 2 : Measure minimum and maximum diameter from our model

To begin, based on our optical design and camera specifications, we establish the relationship between our image pixels and their actual size in millimeters:

$$\Rightarrow \begin{cases} 2092 & \text{pixels} & = & 1.020 & \text{mm} \\ 926 & \text{pixels} & = & 0.428 & \text{mm} \end{cases}$$

In order to scale our model, we obtained measurements of the ring's width at various points around its circumference, with a 20°interval. All measurements were conducted using MeshLab, and the calculated average width is as follows:

$$\text{Width}_{\text{model}} = 2.80 \pm 0.04 \text{ u.m}$$

Given that the width of our model matches the width of our image, we can establish the following ratio between the dimensions of our model and the actual size in millimeters: (where u.m represents the unit of measurement in MeshLab).

$$\text{Width}_{\text{model}} = 2.80 \pm 0.04 = 0.428 \text{ mm}$$
$$\Longleftrightarrow$$
$$1 \text{ u.m} = 0.153 \pm 0.014 \text{ mm}$$

Subsequently, diameters of our model were measured at multiple locations along its circumference, at intervals of 10 degrees. We recorded both the smallest and largest diameters observed:

$$\begin{cases} \text{Diameter}_{\text{min}} & = & 54.04 & \text{u.m} \\ \text{Diameter}_{\text{max}} & = & 54.95 & \text{u.m} \end{cases}$$
$$\Longleftrightarrow \begin{cases} \text{Diameter}_{\text{min}} & = & 8.268 & \text{mm} \\ \text{Diameter}_{\text{max}} & = & 8.407 & \text{mm} \end{cases}$$

While this details the calculations for Ring 1, the exact same method was applied to Ring 2.

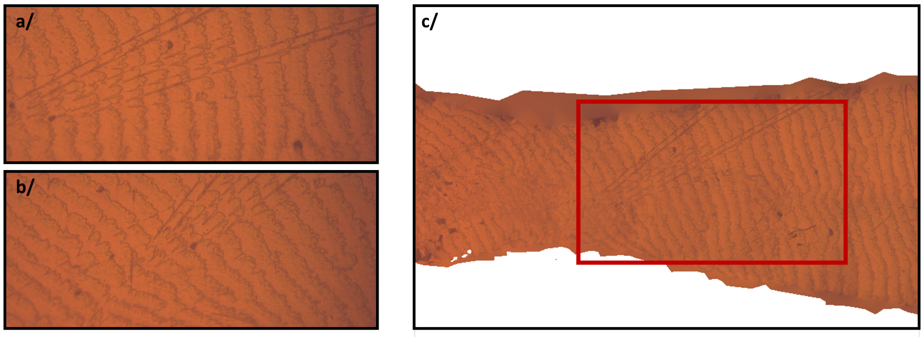

Figure 10: Example of rings intersection in our final model. **a/** Image 1: Acquisition of Ring 1. **b/** Image 2: Acquisition of Ring 2. **c)** A zoomed-in view of the model, highlighting the reconstructed area corresponding to the overlap between Images 1 and 2. This figure effectively demonstrates that our model accurately reconstructs the area where the two rings overlap.
